# Prioritising Responses Of Nurses To deteriorating patient Observations (PRONTO) protocol: testing the effectiveness of a facilitation intervention in a pragmatic, cluster-randomised trial with an embedded process evaluation and cost analysis

**DOI:** 10.1186/s13012-017-0617-5

**Published:** 2017-07-11

**Authors:** Tracey K. Bucknall, Gill Harvey, Julie Considine, Imogen Mitchell, Jo Rycroft-Malone, Ian D. Graham, Mohammadreza Mohebbi, Jennifer Watts, Alison M. Hutchinson

**Affiliations:** 1 Deakin University, School of Nursing and Midwifery, Centre for Quality and Patient Safety Research, Faculty of Health, Geelong, VIC 3220 Australia; 2 Alfred Health, 55 Commercial Rd, Melbourne, VIC 3004 Australia; 3 Adelaide Nursing School, University of Adelaide, Adelaide Health and Medical Sciences Building, Adelaide, SA 5005 Australia; 4 Alliance Manchester Business School, University of Manchester, Manchester, M15 6PB UK; 5 Eastern Health, 5 Arnold St, Box Hill, 3125 VIC Australia; 6 Office of the Dean, Australian National University Medical School, Acton, ACT 0200 Australia; 7 School of Healthcare Sciences, Bangor Institute for Health & Medical Research, Bangor University, Ffriddoedd Road, Bangor, LL572EF UK; 8 School of Epidemiology, Public Health and Preventive Medicine, University of Ottawa, 51 Smyth, Ottawa, ON K1H 8M5 Canada; 9 Faculty of Health, Deakin University, Geelong, VIC 3220 Australia; 10 Centre for Population Health Research, Faculty of Health, Deakin University, Geelong, VIC 3220 Australia; 11 Monash Health, 246 Clayton Road, Clayton, VIC 3168 Australia

**Keywords:** Implementation, Facilitation, Knowledge translation, Clinical decision-making, Patient safety, Guidelines, Vital signs, Randomised controlled trial, Process evaluation, Economic analysis

## Abstract

**Background:**

Vital signs are the primary indicator of physiological status and for determining the need for urgent clinical treatment. Yet, if physiological signs of deterioration are missed, misinterpreted or mismanaged, then critical illness, unplanned intensive care admissions, cardiac arrest and death may ensue. Although evidence demonstrates the benefit of early recognition and management of deteriorating patients, failure to escalate care and manage deteriorating patients remains a relatively frequent occurrence in hospitals.

**Methods/design:**

A pragmatic cluster-randomised controlled trial design will be used to measure clinical effectiveness and cost of a facilitation intervention to improve nurses’ vital sign measurement, interpretation, treatment and escalation of care for patients with abnormal vital signs. A cost consequence analysis will evaluate the intervention cost and effectiveness, and a process evaluation will determine how the implementation of the intervention contributes to outcomes. We will compare clinical outcomes and costs from standard implementation of clinical practice guidelines (CPGs) to facilitated implementation of CPGs. The primary outcome will be adherence to the CPGs by nurses, as measured by escalation of care as per organisational policy. The study will be conducted in four Australian major metropolitan teaching hospitals. In each hospital, eight to ten wards will be randomly allocated to intervention and control groups. Control wards will receive standard implementation of CPGs, while intervention wards will receive standard CPG implementation plus facilitation, using facilitation methods and processes tailored to the ward context. The intervention will be administered to all nursing staff at the ward level for 6 months. At each hospital, two types of facilitators will be provided: a hospital-level facilitator as the lead; and two ward-level facilitators for each ward.

**Discussion:**

This study uses an innovative, networked approach to facilitation to enable uptake of CPGs. Findings will inform the intervention utility and knowledge translation measurement approaches. If successful, the study methodology and intervention has potential for translation to other health care standards.

**Trial registration:**

Australian New Zealand Clinical Trials Registry (ANZCTR), ACTRN12616000544471p

## Background

Contemporary hospitals treat increasingly complex patients suffering from multiple health care problems [1]. Although there are many tools available to assess patients’ responses to illness and treatment, vital signs (VS) are the primary indicator of physiological status and the most common assessment technique employed in health care. If physiological signs of deterioration are missed, misinterpreted or mismanaged, then serious adverse events (SAEs) such as critical illness, cardiac arrest, unplanned intensive care admissions and death may result. Identification of abnormal VS can assist in the detection of at-risk patients minutes to hours before the occurrence of SAEs [2]. VS measurement and escalation of care is fundamental to patient safety and the first step in patient rescue.

International evidence highlights the severity of adverse outcomes when VS are not recognised as abnormal or responded to appropriately [3, 4]. An Australian study found that the presence of three or more abnormal VS was associated with a 19-fold increase in mortality risk as compared to patients with a single abnormal VS [5]. Since 1995, Australia has led the way in system redesign, countering the morbidity and mortality associated with failure to rescue. One strategy, rapid response systems (RRS) and in particular medical emergency teams (MET) takes critical care expertise to the wards when predetermined activation criteria (VS and other measurements) are met. The success of MET and impact on patient outcomes depends on activation actually occurring when indicated. However, we have shown that this does not always occur with 3–53% of patients meeting criteria for activation actually receiving a RRS call [6–8]. Failure to call the RRS for patients fulfilling activation criteria occurs despite the release of international guidelines [9, 10] and the prioritisation of Standard 9 (recognition and response to clinical deterioration in acute health care) as one of the ten National Quality and Safety Health Service (NQSHS) Standards required for Australian hospital accreditation from 2011 [11]. A significant gap between current evidence and practice exists.

In our survey of 1688 patients in 10 acute care hospitals, the prevalence of patients fulfilling MET criteria in the 24 h prior to data collection was 5.3% and on the day of observation was 3.3% [7]. Of the 55 patients who fulfilled the MET criteria, only 2 (3.6%) had a MET call activated within 24 h. Patients who fulfilled MET criteria at the time of vital sign acquisition were more likely to die in hospital (RR = 2.95; 95% confidence interval (CI) 1.22 to 7.15) or within 30 days (RR = 2.64; 95% CI 1.37 to 5.11). Our research has also shown the incidence of patients fulfilling MET calling criteria to be 14.4% during their hospital admission, and of those patients, only 2.9% received a MET call. Further, patients who fulfilled MET activation criteria had double the hospital length of stay [8]. Thus, timely and effective intervention is critical.

Although a growing body of international evidence demonstrates the benefit of early recognition and management of deteriorating patients, suboptimal management has serious consequences and represents a research-practice gap [12]. One recent study further highlighted this gap in a sample of 422 patients; escalation was indicated on 109 occasions, yet contrary to hospital policy, only 58 (53%) were escalated appropriately [6]. This failure to escalate and manage patients concurs with our previous research [7] and others internationally [3, 4, 13]. There is a need to understand the causes, mechanisms and strategies to overcome the problem using knowledge translation (KT) frameworks.

One widely researched and used KT framework is *the Promoting Action on Research Implementation in Health Services* (PARIHS) Framework [14–16], which views implementation as a dynamic and multi-faceted process. The proposition is that successful implementation is a function of the nature and type of evidence, the qualities of the context in which the evidence is being introduced, and the way the process is facilitated [15]. Although many strategies are thought to be effective in promoting individual and organisational change, the change agent role is believed to be a key ingredient for KT. Additionally, systematic reviews have found educational outreach of practice facilitation in health care have improved patient care [17, 18].

## Methods/design

This knowledge translation study uses a pragmatic cluster-randomised controlled trial (C-RCT) design, with process evaluation and cost consequence analysis, to measure the clinical effectiveness and costs of a facilitation intervention to improve nurses’ VS measurement, interpretation, treatment and escalation of patients with abnormal VS. We will compare outcomes and costs from standard implementation of clinical practice guidelines (CPGs) to facilitated implementation of CPGs. An embedded process evaluation will assist in obtaining a deeper understanding of the variable contexts of implementation, the barriers and enablers encountered, the response by stakeholders and the resources required for implementation.

This project aims the following:

1. To improve nurses’ adherence to clinical practice guidelines (CPG) for identifying and managing deteriorating patients in hospital
2. To determine the clinical effectiveness and costs of a **F**aci**l**itation **I**ntervention for **P**ractice improvement (FLIP) to improve nurses’ adherence to the CPGs
3. To assess the impact of contextual characteristics on processes and outcomes of implementation
4. To develop and disseminate recommendations to health policy and practice decision makers

### Primary hypothesis

Nurses in wards receiving standard CPG implementation *plus* a facilitation intervention (FLIP) will demonstrate greater adherence to CPGs for identifying and managing deteriorating patients compared to nurses in wards receiving standard CPG dissemination alone.

### Secondary hypotheses

*Nursing practice outcomes*: Patients in wards receiving the FLIP will have increased documented VS (complete sets), repeated VS within 30 min of obtaining an abnormal VS, documented nursing scope of practice interventions in response to abnormal vital signs, increased activations for urgent clinical reviews and MET calls in patients fulfilling the criteria. *Clinical Outcomes*: Patients in wards receiving the FLIP will have fewer cardio-respiratory arrests, less unplanned intensive care unit (ICU) admissions, lower unexpected hospital mortality rates and reduced hospital length of stay (HLOS). *Health Economic Outcome*: The cost of implementing the FLIP will be offset by savings from shorter HLOS and reduced ICU admissions. FLIP will represent a cost-effective use of resources.

**Fig. 1:**
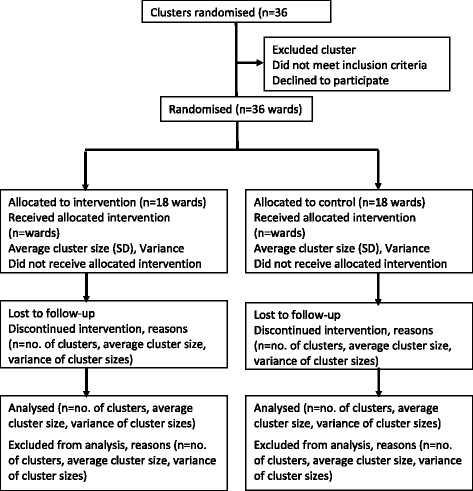
Consort diagram. This figure provides the flow diagram of the phases of the cluster-randomised controlled trial

The study is designed to adhere to the Good Clinical Practice (GCP) Guidelines for national ethics standards and is consistent with the C-RCT CONSORT statement [19] and the CHEERS statement [20], ensuring it meets international ethical and scientific quality standards for reporting clinical trials that include an economic evaluation (see Fig. 1).

### Outcome measures

#### Primary outcome

The primary outcome is the adherence to the CPGs for recognition and management of clinical deterioration by nurses, as measured by the escalation of care as per hospital policy.

#### Secondary outcomes

The secondary outcomes are as follows: *Nursing practice*: Nurses’ adherence to CPGs measured by proportions of documented VS (complete set), repeated VS within 30 min of obtaining an abnormal VS and documented nursing scope of practice interventions in response to abnormal vital signs; proportions of activated urgent clinical reviews and MET calls in patients fulfilling the criteria. *Clinical:* Rates of cardio-respiratory arrests, unplanned admissions to ICU, unexpected hospital mortality and mean HLOS. *Costs:* Mean additional cost of implementing the intervention and mean/median total hospital cost and HLOS for patients. *Process evaluation*: measurement of the fidelity of the facilitation intervention, contextual influences and impact on ward processes and practice.

### Setting

Four hospitals in Victoria, Australia, will be the setting for this study. All are acute, major metropolitan teaching hospitals that have more than 400 beds, employ over 1300 nurses and cover both acute and specialist services. Sponsorship for the study was sought from the Executive Directors of Nursing and Chief Executive Officers of each hospital prior to the funding application. Within each hospital, eight to ten wards will be randomly allocated to either intervention or control groups; the intervention will be delivered at ward level. Each ward is considered a cluster for the purpose of analysis. Wards will be eligible for inclusion if they contain 18 or more beds and they receive support from the hospital’s MET. Critical care, emergency, paediatrics, maternity, peri-operative and psychiatric areas will be excluded because they use an alternative response system for patient deterioration.

### Sample/participants

All staff working within study wards and whose role is direct care delivery will be involved in the study. Each ward has a permanent staff, reducing the likelihood of contamination from staff movement. Nurse managers (NM) will recruit staff to participate in focus groups or individual interviews for the process evaluation; the RA will obtain consent. Medical records of patients from participating wards will be audited over three randomly selected 24-h periods during 1 week at three time points (baseline, immediately post-intervention and 6 months post-intervention) to identify all ward patients in a 24-h period who have abnormal VS. These cohorts of patients will be followed up for the duration of their hospital stay and analysed according to ward (intervention or control), irrespective of subsequent ward transfers that may occur using the principles of intention-to-treat (ITT) analysis.

### Sample size

From a medical record review in one of the sites, the rate of adherence to escalation policy after abnormal VS measurement was 53% [6]. Sample size calculations are based on a 50% rate of adherence to escalation as per hospital policy. To detect a 20% improvement in adherence rate in the intervention group immediately following and 6 months post-intervention, 270 documented abnormal VSs per time point per study arm are required. Our sample size has been inflated to allow for a design effect of 1.5, accounting for within-patient autocorrelation and within ward clustering effect. Type I error is set at a 1% significance level accounting for multiple comparisons and study power is set to 90%. For secondary outcomes, we have 80% power to detect an odds ratio of at least 1.8 in post-intervention comparisons for an improved adherence rate in the intervention compared to the control group. For all other secondary outcomes, power calculations have been based on combining immediately following intervention and 6 months post-intervention data. For the unplanned ICU admission rate, a minimum of 0.3% difference (1.2 vs 0.9%); for MET call rate a minimum of 2.3% absolute difference (3 vs 0.7%); and at least 1.6 day (IQR = 20) median absolute difference (12 vs 10.4) for hospital LOS is detectable at 80% power. Due to a low incidence of cardio-respiratory arrests and hospital mortality, a power analysis is based on combining mortality and code blue, resulting in 80% power to detect a minimum 2.3% absolute difference (3 vs 0.7%).

### Randomisation

Random 1:1 block randomisation of wards within hospitals to intervention or control groups will be undertaken. A central randomisation service independent of the study will be used with concealment until the intervention is assigned. Assessment days will be randomly selected.

### Control group

The control group ward staff will receive the standard implementation of CPGs. This will include information on the CPG, its availability online, as well as notification of the free online educational courses available to increase their knowledge and understanding about the identification and management of deteriorating patients. Information will be provided to each NM for dissemination to ward staff.

### Intervention group

The intervention group ward staff will receive the standard implementation of CPGs as described above plus the facilitation intervention for practice improvement (FLIP). FLIP comprises individuals in facilitator roles using facilitation methods and processes in a flexible way to tailor implementation strategies to the local ward context, according to identified barriers and enablers.

There will be two types of internal facilitator roles: hospital facilitators (HFLIP) and ward facilitators (WFLIP). A hospital facilitator will be appointed to cover four or five intervention wards in each of the four hospitals, acting as the overall facilitation lead and coordinator and mentor for the ward facilitators in each hospital. In each intervention ward, two ward facilitators will be appointed to support and enable ward nurses to implement the CPGs into practice (*N* = 36). Each HFLIP will be prepared and supported by an external expert in facilitation (GH). Each HFLIP will work with eight to ten WFLIPs. The facilitation intervention is underpinned by the PARIHS [14, 16] and i-PARIHS [21] frameworks.

Training and a toolkit of methods and techniques will be given to all FLIPs to promote the transfer of CPGs into daily practice. The intervention wards will receive 5 h per week support from the HFLIP for 6 months, reviewing barriers and enablers to implementing CPGs into practice prior to intervention commencement and in discussion with the WFLIPs. The HFLIP will provide face to face interactive education, including small group work, case presentations and role play, as well as review ward specific systems, processes, routines and audit practices in order to align systems of care to enable implementation of CPGs into daily practice. At ward level, the two WFLIPs will work with the HFLIP to audit practice, identify barriers, strategies and goals for improvement. They will provide cover for each other during leave and support ward staff during practice hours, receiving one day per fortnight of protected time for conducting audits, facilitation of ward nurses and meeting with the HFLIP to agree on strategies to improve adherence to guidelines.

### Recruitment and selection of facilitators

HFLIP positions will be advertised on the hospitals’ intranet and through a snowballing technique, to identify interested staff. Local staff will be interviewed and selected to build capacity within the participating organisations at a clinical leadership level. HFLIPs should have some prior experience (e.g. in practice development or an education role) so that they can support and mentor the WFLIPS and deal with the more challenging contextual barriers that may arise. Selection of all FLIPs will be based on the following characteristics: knowledge of and interest in NSQHC Standard 9; knowledge of colleagues, ward, and organisation; holds a clinical leadership role; and possesses good communication skills. Nurse managers will be asked to identify two WFLIPs for their wards based on the above criteria. All potential WFLIPs will be asked to consent to participation.

### Training and intervention fidelity

Research assistants (RAs) and facilitators will undergo onsite training to ensure consistency across all sites. Training will be tailored to the type of RA (outcome, process or economic assessor) or facilitation role (WFLIP or HFLIP) and be focused on consistency/fidelity. Standardised procedure manuals will be provided to all RAs and facilitators with specific detail on roles and responsibilities.

HFLIPs will receive training for 3 days from two experts in recognition and response to deterioration and advanced facilitation skills. HFLIPs will receive mentoring and ongoing support throughout the trial in a monthly teleconference with an external facilitator and Project Manager, as well as opportunities to teleconference at any time should questions or issues arise. One face to face HFLIP meeting and site visit will be conducted during the intervention. HFLIPs will receive contact details for other HFLIPs to encourage communication across sites, share learnings and troubleshoot barriers to facilitation. Ward FLIPs will receive training for a single day including knowledge on recognition and response to deterioration, beginner facilitation skills, and will receive mentoring and ongoing support during the trial. Support for WFLIPs will be provided by HFLIPs during the trial in the time allocated to each ward and via phone or email at other times. All interactions with WFLIPs will be captured by HFLIPs using electronic activity logs. Education modules, developed and used previously in research will be employed, including the following: using evidence in professional practice, implementing guidelines into practice, identifying and managing deteriorating patients, and facilitating and auditing. The intensity of the intervention will be monitored across sites. All HFLIP activities will be recorded on electronic activity and communication logs. HFLIPs will also audit ward practice 1 week each month for the duration of the intervention period. Audits will include half the occupied beds in each ward (every second bed). Audit data will be collected electronically and then formatted into feedback graphs and text. Text will contain a main message arising from the data. Feedback will be delivered to each WFLIP for discussion at ward meetings or individually to ward staff. WFLIPS will be taught the audit process to assist with their understanding of ward practices in relation to recognition and management of patient deterioration and for practice sustainability at the completion of the trial.

Research assistants performing data extraction from medical records will be given training on the electronic data collection tool and accompanying data dictionary. Inter-rater reliability testing will be established by independent double auditing across sites until Kappa >0.95 is achieved, with a minimum of 10 medical records audited per research assistant. In addition, a study monitoring audit will be conducted by the project manager on a random sample of 100 patient charts across four sites to ensure continued accuracy of data extraction by research assistants.

### Blinding

All RAs will be blinded to group allocation. Success of blinding will be assessed at study end using the James Blinding Index [22]. Although facilitators will not be blinded and nurses will be aware of the FLIP, ward nurses will be unaware of the comparator. Patients will be unaware of the intervention. MM, JW and the RAs will analyse the data, and all will be blinded to group allocation.

### Instruments

The electronic Case Report Form (eCRF) was adapted from our previous research [6, 7] to collect data relating to (1) documented vital signs (respiratory rate, heart rate, temperature, blood pressure, conscious state and oxygen saturation), CRC and MET activations; (2) patient outcomes, including unplanned ICU admissions, cardio-respiratory arrests, limitations of medical treatment orders, LOS and inpatient mortality; and (3) nursing interventions and referrals in response to abnormal vital signs and escalation as per hospital policy.

Electronic Activity and Communication Log (eACL) data will be collected at the time of activity and throughout the duration of the intervention. Data captured will include methods and techniques outlined in the Facilitator Manuals, enablers and barriers, and measures of impact.

### Assessments

#### Aims 1 and 2

Outcome data collection will occur across three time points: at baseline prior to implementation, immediately following completion of the intervention (6 months) and 6 months post-intervention (12 months). At each time point, the medical records of all inpatients admitted to study wards will be reviewed by a RA (blinded to group allocation) to determine if abnormal VS were present, and if so, the rates of escalation as per hospital policy, HLOS, unplanned ICU admissions and unexpected inpatient mortality. Data collection days will be randomly selected. RAs will be trained in data entry. Prior to RAs collecting data independently, inter-rater reliability will be evaluated by the project manager who is a registered nurse with specialist intensive care qualifications. Independent data extraction will be conducted until >95% agreement is obtained. The secure data entry system will not allow invalid values; data backup measures will be installed. Costs attributed to FLIP and training will be calculated.

#### Aim 3

Facilitators will complete electronic activity logs; ward communication and policy changes will be collected by RAs electronically. To capture the experience of FLIPs and key stakeholders regarding the intervention and CPG implementation, semi-structured individual and focus group interviews and field observations will be conducted at critical points in the project—before, during and after the intervention. Interviews will be audio-recorded and transcribed verbatim.

All assessments are outlined in Table 1.

**Table 1:** Data collection

Aim	Variable/Measure	Instrument	Outcomes	Data collection point
1	Adherence to CPG	eCRF	CPG element compliance: completed VS documentation; repeated VS within 30 min; interventions within scope of practice; CRC and MET activations; escalation as per hospital policy.	Baseline^a^, 6 and 12 months^b^
2	Patient outcomes	eCRF; Hospital clinical databases	Unplanned ICU admissions, cardio-respiratory arrests, limitations of medical treatment orders, HLOS and inpatient mortality.	Baseline^a^, 6 and 12 months^b^
2	Hospital costs	CRF; Hospital clinical costing databases.	Unplanned ICU admissions, HLOS and inpatient mortality; total cost of hospital episode.	Hospital episode^b^
2	Intervention costs	CRF; Staffing salaries	FLIP cost; training frequency	Continuous
3	FLIP dose	eACL	Contact and content of contact.	Continuous
3	Enablers/barriers	eACL/ Ward Communication Books, RISKMAN incidents, Focus groups.	Issues arising; Critical incident analysis.	Continuous
3	Ward Impact	Ward Communication Books, policies and procedures, interviews, field observations.	Frequency and content.	Continuous
3	Facilitator Impact	Interviews with all FLIPS	Perceptions and content.	6 months^b^

^a^Prior to commencement of intervention (baseline)

^b^On completion of intervention (6 and 12 months post-baseline data collection)

### Data analysis

Quantitative methods will be used to analyse patient, health services utilization and economic outcomes. Main and secondary outcome analysis will be based on all randomised wards and selected participants (intention-to-treat analysis). To account for within-patient correlation, due to multiple measurements from the same patient during assessment days, we will implement generalized estimating equation (GEE) models with binary outcome and logit link for all rate outcome comparisons [23]. The Cochran-Mantel-Haenszel (CMH) test will be used to compare proportions. Clustering effects for wards will be evaluated by calculating intraclass correlation coefficients and, if necessary, a fixed effect nominal factor will be included in GEEs to account for ward impact. A series of exploratory analyses will be conducted on subgroups and the impact of covariates on estimates of the effect of the intervention. A nonparametric median test will be used for HLOS comparison. In supportive analysis, HLOS will be considered as time to event data. Survival rates will be calculated and illustrated by the Kaplan–Meier method and further analysed by the log-rank test for univariate analysis (stratified by wards). Variables that reveal prognostic or effect modifying potential on the outcome as suggested by univariate analysis will subsequently be evaluated by the proportional Cox regression for multivariate analysis. Hazard ratios with the corresponding 95% confidence intervals will be reported. A *p* value of <0.05 is considered statistically significant. Data will be analysed using Stata version 14 or later [24].

#### Cost analysis

Analysis of the costs of hospitalisation will be determined at the patient level over the 6-month period for intervention and control wards. All patients admitted to the study wards during the 6-month study period will be included in the analysis of mean total cost, HLOS and ICU LOS for intervention and control wards in each hospital. Only complete admissions will be included (patients admitted to a study ward and discharged from the hospital within the study period); and patients will be analysed based on their admission ward. Mean costs and HLOS for each patient in each ward will be compared to costs and HLOS for patients admitted to the same wards in the 6 months prior to the study period. Difference in difference analyses will be undertaken to compare the mean cost and HLOS difference between pre-intervention and intervention periods, and between intervention and control wards across hospitals. Costs will be attributed to the FLIP intervention based on the additional costs of the implementation process (staff time) over standard in-hospital CPG training.

#### Process evaluation

In order to determine how the implementation of the intervention contributes to the observed outcomes, data will be collected in four domains: intervention implementation, the recipients of the intervention perceptions and views of the evidence and implementation, responses of the staff implementing (or supporting implementation) of the CPG recommendations and the contextual influences mediating implementation. Interviews, field observations and focus group transcripts will be subject to thematic analysis [25]. Analysis will be iterative, refining emerging themes, then comparing themes and relationships through a process of pattern matching to examine for data consistency. Themes and relationships will be re-examined and recoded by two members of the research team. Analysis will continue until no new themes emerge and agreement on themes is achieved.

### Develop and disseminate recommendations with health policy and practice decision makers

#### Aim 4

An expert advisory group (EAG) consisting of Chief investigators, Associate Investigators and Partner Organisation representatives with expertise in nursing, medicine, NSQHS Standard 9, KT and policy making will develop recommendations that bring together key elements, strategies, tools and resources identified to improve patient safety and provide recommendations for system-wide health service improvements.

We will use a multi-faceted approach to translate and disseminate the findings of this study to health professionals, administrators and policy-makers. The study methodology and findings will be of particular interest and relevance to health professionals and administrators. The findings will also be of interest to those wanting to better understand how to translate evidence into practice through facilitation. We will use traditional approaches to knowledge dissemination through high-quality publications in peer-reviewed journals and presentations at major international and national conferences, as well as through our formal and informal networks, nationally and internationally. All members of the expert advisory group have active collaborations with other health care organisations and National Advisory Committees. Forums and presentations will be held at participating hospitals which are committed to knowledge sharing in order to promote safe, high-quality care delivery.

## Trial status

This study has received ethics approval and data collection is currently underway.

## Discussion

Individual patients vary in their responses to treatment, and their health status can change rapidly with little warning. Critical illness, cardiac arrest, unplanned intensive care admissions and death may result if physiological signs of deterioration are missed, misinterpreted or mismanaged. These serious events occur multiple times daily in hospitals and frequently result from communication failures during changes in a patient’s clinical status. The consequences for the patient can be catastrophic and the costs to the community are significant [13].

Despite the existence of international guidelines that recommend early recognition and response to deterioration [10, 11, 26], a research-practice gap remains. Much of the focus has been on disseminating decision support tools in organisations rather than enabling change in clinician behaviour. These factors along with calls for more theory-informed interventions, using robust and methodologically sound research [27], have prompted the development of this facilitation intervention. The study design focuses on testing the effectiveness of a facilitation intervention to implement clinical practice guidelines on the recognition of and response to clinical deterioration by ward nurses. This study evaluates a networked approach to facilitation, creating an organizational infrastructure and building capacity in the skills and knowledge required for translating evidence into clinical practice. Facilitators use facilitation skills and knowledge to help individuals, teams and organisations apply evidence into practice [15]. This research will investigate the relationship between facilitation and the impact on evidence use and patient outcomes, as well as contextual factors that influence implementation processes.

This study uses an innovative and novel approach to deliver a networked approach to facilitation to enable knowledge translation. It will inform knowledge translation measurement approaches using process evaluation and health economic techniques. If successful, the facilitation methodology has the potential for translation to other health care standards such as medication safety, falls and pressure injury prevention; all high-risk areas for health services and important issues for patients and their families.

## Abbreviations

ANZCTR: Australian New Zealand Clinical Trials Registry
CHEERS: Consolidated health economic evaluation reporting standards
CMH: Cochran-Mantel-Haenszel
CPGs: Clinical practice guidelines
C-RCT: Cluster-randomised controlled trial
eACL: Electronic activity and communication log
EAG: Expert Advisory Group
eCRF: electronic case report form
FLIP: **F**aci**l**itation **I**ntervention for **P**ractice improvement
GCP: Good clinical practice
GEEs: Generalized estimation equation models
HFLIP: Hospital facilitator
HLOS: Hospital length of stay
ICU: Intensive care unit
ITT: Intention to treat
KT: Knowledge translation
MET: Medical emergency teams
NMs: Nurse managers
NQSHS: National Quality and Safety Health Service
PARIHS: Promoting Action on Research Implementation in Health Services
PRONTO: Prioritising Responses Of Nurses To deteriorating patient Observations
RAs: Research assistants
RRS: Rapid response systems
SAEs: Serious adverse events
VS: Vital signs
WFLIP: Ward facilitator
